# Influence of Interactions Between Drawing Soy Protein and Myofibrillar Proteins on Gel Properties

**DOI:** 10.3390/foods14061064

**Published:** 2025-03-20

**Authors:** Tong Jiang, Yujie Zhao, Mingming Huang, Zhiyong Zhang, Yanwei Mao, Huixin Zuo

**Affiliations:** 1 College of Food Science and Engineering, Shandong Agricultural University, Tai’an 271018, China; 13064653588@163.com (T.J.); zhaoyujie2001@163.com (Y.Z.); hades3709@126.com (M.H.); maoyanwei@sdau.edu.cn (Y.M.); 2 Tongliao Academy of Agricultural and Animal Husbandry Sciences, Tongliao 028015, China; dalanpisu@163.com

**Keywords:** drawing soy protein, myofibrillar proteins, gels, water-holding capacity

## Abstract

Drawing soy protein (DSP) exhibits a well-defined fibrous structure, conferring significant market potential. This study investigates the interactions between DSP and myofibrillar proteins (MP) and their effects on gel properties. Porcine myofibrillar protein (MP) was used as the raw material, and mixed systems were prepared by incorporating different concentrations of DSP at 0%, 2%, 4%, 6%, and 8% to evaluate their physicochemical properties and gel characteristics. The results demonstrated that the addition of DSP enhanced the gel strength, hardness, and water-holding capacity (WHC) of MP, thereby improving the overall properties and water retention of the gels. Among them, the trend of change was most obvious when the addition amount was 6%. The gel strength increased by 196.5%, the water retention capacity improved by 68.3%, and the hardness rose by 33.3%. Furthermore, as the addition amount of DSP increases, the total thiol content decreases, the hydrogen bond content increases, and the surface hydrophobicity enhances. This leads to a more compact arrangement of protein molecules, which is conducive to a denser and more stable solution and improves the stability of the protein solution. The α-helical structures in the proteins progressively transformed into β-turn structures, exposing more amino acid side chains and inducing conformational changes in MP, resulting in denser and more uniform gel network structures. The most pronounced changes were observed at a 6% addition level. These findings contribute to diversifying meat products and provide a theoretical basis for improving the WHC and yield of emulsified meat products in pork processing.

## 1. Introduction

In recent years, pork products have occupied a significant market development space due to their unique nutritional and flavor characteristics, attracting considerable attention to the functional properties of pork proteins. Myofibrillar protein (MP), the primary functional protein in muscle tissue, plays a crucial role in gel formation [1]. MP is a complex composed of myosin, actin, actomyosin, tropomyosin, and troponin, among others [2]. The extraction process entails denaturing the spatial structure of MP at elevated temperatures, followed by aggregation at lower temperatures via intermolecular forces including hydrophobic interactions, hydrogen bonding, electrostatic attractions, and disulfide linkages, ultimately resulting in an organized three-dimensional network structure [3]. Consequently, the characteristics of thermally induced gels significantly influence the flavor, texture, water-holding capacity (WHC), and yield of meat products [4].

Gel performance is one of the most critical properties for evaluating the quality of comminuted meat products. According to existing research, various factors affect the gel properties of these products, including the type of meat, production processes, and exogenous additives. Among these, exogenous additives are commonly used to enhance the gel strength of comminuted meat, which includes exogenous additives such as animal and plant proteins, starches, hydrocolloids, and other natural products [5]. Plant proteins, in particular, are widely utilized in comminuted meat processing to improve nutritional value. Borderías et al. [6] found that adding an appropriate amount of pea protein isolate can form myofibrillar protein gels with a well-linked network and enhanced health benefits. Li et al. [7] demonstrated that treating porcine MP with different concentrations of soy protein isolate under high pressure significantly affected WHC, rheological properties, and gel characteristics. Lin et al. [8] observed that mixtures of soy protein isolate and peanut protein isolate, as well as soy protein isolate and rice protein isolate, could improve the gel characteristics of red snapper MP.

With the improvement in people’s quality of life, traditional soy protein isolates can no longer fully meet consumers’ sensory demands. Therefore, food researchers have developed drawing soy protein (DSP) to satisfy consumer preferences for texture and taste [4]. DSP is a structured protein made from ingredients such as soy protein isolate and soy protein concentrate, processed using extrusion technology. This structured protein exhibits a rich fibrous structure, providing a chewing sensation similar to meat and enhancing characteristics such as oil absorption, chewiness, and water retention. Compared to traditional animal proteins, DSP prepared by extrusion and puffing does not contain cholesterol and offers advantages of being low in fat and calories, making them a high-quality, healthy food option [9]. Moreover, with its fibrous protein structure and meat-like texture, this plant protein has substantial market potential [10].

Research has demonstrated that DSP is capable of interacting with MP to create a tightly woven three-dimensional network structure, thereby enhancing the gel strength of comminuted meat products [11]. However, there is currently limited research on the mechanisms underlying the interaction between soy proteins and MP to improve gel characteristics. Therefore, this study investigated the interactions between varying amounts of DSP and extracted porcine MP, as well as their impact on gel properties, aiming to provide a reference basis for producing high-quality pork comminuted meat products.

## 2. Materials and Methods

### 2.1. Materials

The porcine longissimus dorsi used in this experiment was purchased from a fresh convenience supermarket in Tai’an City, and the DSP was procured from SuLian Food Co., Ltd. in Ningbo City (Ningbo, China).

### 2.2. Extraction of MP

Based on the method of Jiang et al. [12], 10 g of frozen and minced pork was homogenized with 50 mL of 4 °C isolation buffer (10 mM phosphate, 0.1 M NaCl, 2 mM MgCl_2_, and 1 mM EGTA, pH 7.0) at 12,000 r/min for 30 s. The muscle homogenate underwent centrifugation at 2000× *g* for 15 min at a temperature of 4 °C, after which the supernatant was removed. The pellet was rinsed twice with the same buffer, maintaining consistent mixing and centrifugation conditions, using over 40 mL of buffer per wash. Subsequently, the myofibrillar pellets were rinsed three times using 40 mL of 0.1 M NaCl solution while maintaining the same conditions. After the third wash, the filtrate was passed through four layers of cheesecloth, and the resulting filtrate was adjusted to pH 6.0 using HCl solution. The filtrate was then centrifuged, and the precipitate obtained was identified as myofibrillar protein (MP). Finally, the MP pellets were dissolved in 0.6 mM NaCl (pH 6.5), and the concentration was adjusted to 50 mg/mL. The MP solution was stored at 4 °C and used within 24 h. The concentration of MP was determined by the BCA method.

### 2.3. Determination of Gel Properties

#### 2.3.1. Preparation of Myofibrillar Protein Gels

MP solutions were supplemented with DSP at concentrations of 0%, 2%, 4%, 6%, and 8% (*w*/*v*), respectively. The mixtures were homogenized with a blender at a speed of 6000 rpm for 1 min. Subsequently, the homogenized samples were transferred to 10 mL beakers and heated in a water bath at 80 °C for 30 min to induce gel formation. Following heating, the gels were allowed to cool to ambient temperature and then stored at 4 °C for subsequent analysis.

#### 2.3.2. Gel Water-Holding Capacity (WHC)

With slight modifications to the approach described by Xia et al. [13], 2 g of MP glue were cut into small pieces and placed in a 10 mL centrifuge tube and centrifuged at 9000× *g* for 10 min at 4 °C. After centrifugation, the supernatant was removed, and the weight of the gel was measured. The WHC was calculated as the ratio of the gel’s weight post-centrifugation to its total initial weight, multiplied by 100%. The experiment was conducted in triplicate.

#### 2.3.3. Gel Strength

Based on the method described by Fowler et al. [14], cylindrical samples (40 mm in diameter × 20 mm in height) of porcine MP-DSP composite gel were subjected to texture profile analysis using a texture analyzer (TA-XT2i, Stable Micro System, Godalming, UK). Measurement parameters were set as follows: a P/5S probe was used, with a deformation level of 40%, a trigger force set at 5 g, a pre-test speed of 2 mm/s, and both the testing and post-test speeds maintained at 1 mm/s.

#### 2.3.4. Texture Profile Analysis (TPA)

The MP gels were cut into cylindrical sections with a height of 2 cm and then subjected to TPA testing using a texture analyzer. Key experimental parameters included a P 50 probe and a 50% compression ratio. The pre-test speed was set to 2.00 mm/s, the test speed to 1.00 mm/s, and the post-test speed to 5.00 mm/s. The displacement was 1.00 mm, and the trigger force was set at 5 g.

#### 2.3.5. Scanning Electron Microscopy (SEM) Examination

The microstructure of MP gels was determined following the method described by Zhang et al. [15]. The MP gel samples were cut into small strips and immersed in 2.5% glutaraldehyde (pH = 6.8, dissolved in 10 mmol/L phosphate buffer) for 24 h at 4 °C for fixation. They were then rinsed with 0.1 mol/L pH = 6.8 phosphate buffer. The samples were successively washed with 30%, 50%, 70%, 80%, 90%, and 100% ethanol solutions for 10 min each time for dehydration treatment. Each group was repeated three times. The samples were then soaked in tert-butanol for 10 min to replace the ethanol (repeated 3 times, each for 15 min). After freeze-drying, the MP gel samples were mounted on an SEM sample stage and sputter-coated with gold for examination. The specimens were examined under a scanning electron microscope at a magnification of 20,000× with an accelerating voltage of 5.0 kV.

### 2.4. Total Sulfhydryl Content

The determination of total mercaptan content was carried out by the Ellman method. Specifically, 0.1 mL of MP solution was added to 0.9 mL of phosphate-buffer solution containing urea (50 mM phosphate buffer, 8 M urea, 10 mM EDTA, 0.6 M potassium chloride, pH = 7.0), and 0.04 mL of DTNB solution. For the control group, the MP solution was replaced with a 20 mM phosphate-buffer solution (20 mM phosphate buffer, 0.6 M sodium chloride, pH = 6.5). After mixing, the solution was incubated in the dark at 37 °C for 30 min, and the absorbance was measured at 412 nm with a molar absorption coefficient of 13,600 M^−1^ cm^−1^. The total sulfhydryl content was calculated according to the Lambert–Beer law, and the results were expressed as the amount of total sulfhydryl per gram of meat sample. The experiment was repeated three times.

### 2.5. Surface Hydrophobicity

The determination of surface hydrophobicity was based on the method of Chelh et al. [16] with minor modifications. In 1 mL of 2 mg/mL MP solution, 40 μL of 1 mg/mL bromophenol blue solution was added and mixed at room temperature for 15 min. After the reaction, the mixture was centrifuged at 3000× *g* for 15 min at 4 °C, and the supernatant was collected to measure the absorbance at 595 nm. A control group was prepared by replacing the MP solution with 1 mL of 20 mmol/L phosphate-buffer solution (0.6 mol/L NaCl, pH = 6.5). The surface hydrophobicity index was characterized by the amount of bromophenol blue bound, calculated using the following formula:

$$Bromophenol\;blue\;binding\;amount\left(\mu g\right)=40\;\mu g\times \frac{A\;control\;group-A\;samples}{A\;control\;group}$$

### 2.6. Rheological Properties

The determination of rheological properties was modified according to the method of Cao et al. [17]. When conducting the measurement using the modular intelligent rheometer (Aton Paar, Graz, Austria), a small-amplitude oscillation temperature program was adopted. A stainless steel parallel plate probe with a diameter of 40 mm was used, and the gap between the probe and the plate was 1.0 mm. Five g of the MP-DSP mixed gel was evenly spread between the two plates, and a thin layer of paraffin oil was applied on the edge of the plates to prevent dehydration at the sample edge and evaporation of water in the sample. After equilibrating at an initial temperature of 25 °C for 3 min, the sample was heated continuously from 25 °C to the final temperature of 80 °C at a heating (cooling) rate of 3 °C/min. During the heating process, a dynamic temperature scan was performed at an angular frequency of 95 r/min and a strain of 0.1% to obtain linear responses within the viscoelastic region. After setting the parameters, the changes in storage modulus (G′) and loss modulus (G″) of the sample with increasing temperature were measured.

### 2.7. Intermolecular Interaction Forces

A total of 2 g of chopped mixed gel samples was weighed and separately mixed with 10 mL of 0.05 mol/L NaCl (A), 0.6 mol/L NaCl (B), 0.6 mol/L NaCl + 1.5 mol/L urea (C), and 0.6 mol/L NaCl + 8 mol/L urea (D). The mixtures were homogenized and let to stand at 4 °C for 1 h. The mixtures were centrifuged at 8000 r/min and 4 °C for 20 min, and then the protein concentration of the supernatant was measured using a BCA assay kit. The differences in protein concentration between solutions B and A, C and B, and D and C represent the content of ionic bonds, hydrogen bonds, and hydrophobic interactions, respectively.

### 2.8. Raman Spectroscopy Scanning

Raman spectroscopy measurements were conducted based on the method of Berhe et al. [18] with some modifications. For the extracted MP, a laser confocal micro-Raman spectrometer was used to measure the samples. The extracted MP was placed on a microscope slide wrapped with aluminum foil and positioned under the microscope. A 10× objective lens was used to collect the Raman spectra of the samples. The laser excitation wavelength was set to 532 nm, with the laser focused on the sample. The laser parameters were set to a power intensity of 10.7 mW, and the sample was scanned 40 times with an exposure time of 0.05 s per scan. The range of Raman shift acquisition was from 400 to 2000 cm^−1^, and 6 spectra were collected for each sample.

### 2.9. Data Analysis

With varying amounts of DSP as the fixed factor and replication as the random factor, data were subjected to significant analysis using Duncan’s test with IBM SPSS Statistics 26.0 software. Data plotting was performed using Origin 2022 software. Raman data were preprocessed with Origin 2022, and spectral fitting analysis was conducted using PeakFit4.

## 3. Results and Discussion

### 3.1. Analysis of Gel Water Retention

**Figure 1:**
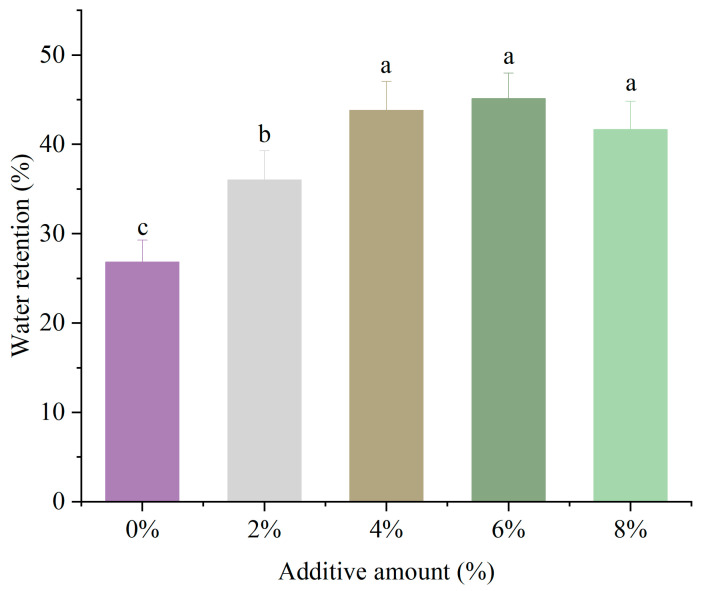
Effect of different DSP addition on the water retention of MP gel. a–c Different letters indicate significant differences within the different addition amounts (*p* < 0.05).

WHC is a primary criterion for evaluating the quality of protein gels. Higher water retention indicates that the gel has a stronger ability to retain internal water and exhibits a denser network structure [19], as shown in Figure 1. Effect of different DSP additions on the water retention of MP gel: the WHC of the gel increased significantly with the addition of DSP (*p* < 0.05), reaching a maximum value at a 6% addition level. This increase may have been due to the exposure of hydrophobic residues in the protein after the addition of DSP, which was conducive to forming a better thermal gel network structure and enhancing WHC [7,19]. Li et al. [20] reported that the addition of SPI could make the protein structure more uniform and the pores smaller, thereby preventing water loss, which was consistent with the results of this study. At the same time, the exposure of hydrophobic residues in the protein and their mutual reactions after the addition of soybean textured protein were beneficial for forming a thermal gel network structure, improving the gel’s WHC. However, when the soy protein addition level reached 8%, the WHC of the gel showed a downward trend. This decline suggests that an excess of protein may lead to an overly dense network structure, hindering water molecule migration and thus reducing the gel’s WHC [7].

### 3.2. Gel Strength

**Figure 2:**
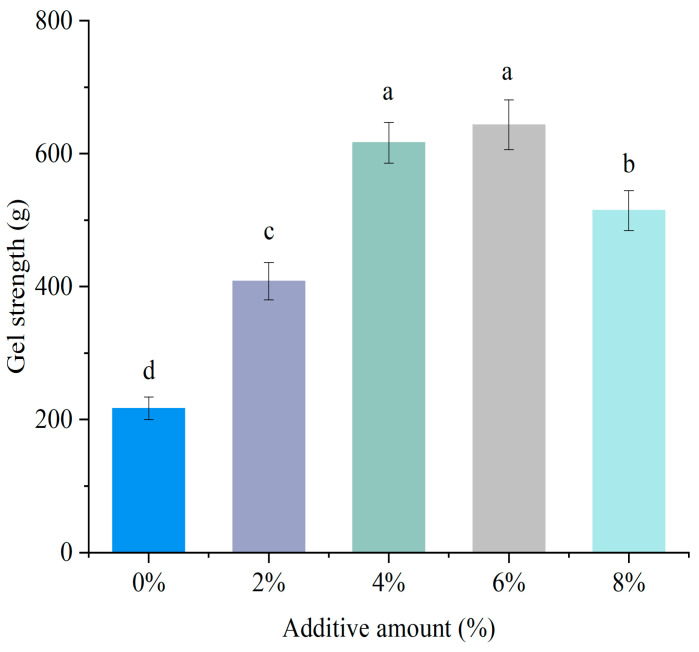
Effect of different DSP additions on the gel strength of MP. (a–d) Different letters indicate significant differences within the different addition amounts (*p* < 0.05).

Gel strength reflects their ability to form cohesive network structures [21]. As could be seen from Figure 2, within the range of 8% soybean textured protein addition, the gel strength of MP showed a trend of first increasing and then decreasing with the increase in DSP addition. The gel strength was highest at a 6% addition level, significantly increasing compared to the control group (*p* < 0.05), indicating that an appropriate amount of soybean textured protein could significantly enhance the gel strength of MP. This might be attributed to the filling effect of DSP. When DSP is embedded in the MP gel network, it fills the gel pores and reduces water migration, thereby making the MP gel network more compact [22]. However, when the addition of DSP reached 8%, the gel strength significantly decreased, indicating that an excessive amount of DSP might hinder the cross-linking of porcine MP, leading to a decline in the overall gel properties of the mixed proteins [8]. Wu et al. [23] reported that a higher ratio of 11S in the mixture leads to aggregates with larger sizes, higher surface hydrophobicity, and more disulfide bonds, thereby forming a porous network with a higher storage modulus, which enhances gel strength. This is consistent with the results of this study.

### 3.3. TPA

Texture, as one of the key factors for evaluating the quality of food, reflects the interactions between proteins and proteins, as well as between proteins and water molecules in gels [24]. Table 1 shows the effects of different doses of DSP on the properties of gels, including changes in hardness, elasticity, texture, adhesiveness, chewiness, and resilience. Without the addition of DSP, the hardness of the MP gel was poor. As the addition level of DSP increased, the hardness of the MP gel significantly increased (*p* < 0.05), reaching a maximum value of 71.48 g at 6%, after which it decreased. Compared to the control group, the adhesiveness and chewiness also followed the same trend. Before the addition of 4% soybean textured protein, the improvement in textural properties was mainly related to the water absorption and expansion of soybean textured protein during the heating gel process [23]; after the addition of 4% soybean textured protein, the decline in textural properties may be due to the excessive addition of soybean textured protein disrupting the gel structure, thereby reducing the textural properties of the gel [8]. In summary, when the addition level of soybean textured protein was 6%, the textural properties of the MP gel were the best, which is consistent with the results of gel strength.

### 3.4. Total Sulfhydryl Content

**Figure 3:**
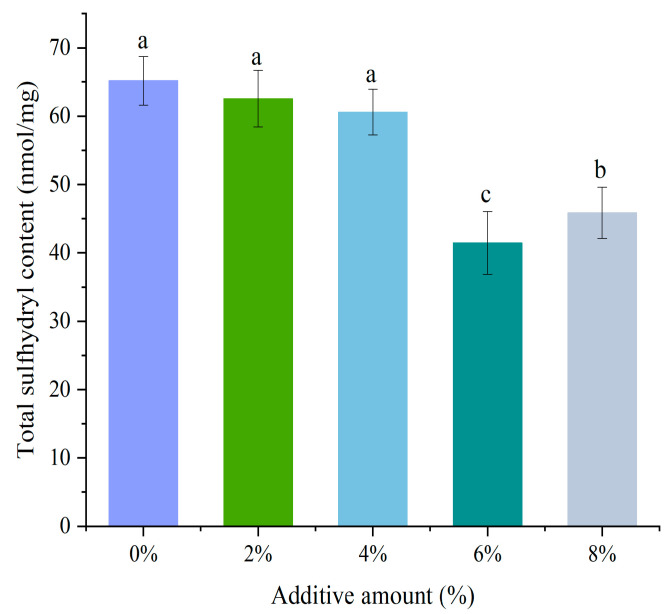
Effect of different DSP additions on the total sulfhydryl content of MP. (a–c) Different letters indicate significant differences within the different addition amounts (*p* < 0.05).

The variation in total sulfhydryl content is closely related to protein denaturation, reflecting changes in the tertiary structure and disulfide bonds of proteins [25]. As shown in Figure 3, with the increase in soybean textured protein addition, the total sulfhydryl content exhibited a decreasing trend. When the addition was within the range of 2% to 4%, the change in total sulfhydryl content was not significant (*p* > 0.05). After the addition of 4%, the total sulfhydryl content significantly decreased (*p* < 0.05), reaching the lowest value at 6%. The main reason for the reduction in total sulfhydryl content is the formation of disulfide bonds between sulfhydryl groups. This change may be attributed to alterations in the spatial structure of MP, leading to the exposure of internal sulfhydryl and their subsequent oxidation into disulfide bonds [26]. In the gel with 8% DSP, there was a notable decrease in the total thiol group content. This reduction could be attributed to excessive physical entanglement between DSP and MP, along with the steric hindrance from DSP that inhibited the unfolding and exposure of thiol groups in MP [15]. Riebroy et al. [25] found in their research that an increase in the hydrophobicity of the surface of cod myosin would lead to the exposure of hydrophobic groups, thereby promoting protein denaturation and aggregation, and further resulting in the formation of disulfide bonds through thiol cross-linking, which is consistent with the results of this study. It is analyzed that when the addition of soybean textured protein is 6%, a large number of sulfhydryl are oxidized to form disulfide bonds, contributing to a more stable solution.

### 3.5. Surface Hydrophobicity

**Figure 4:**
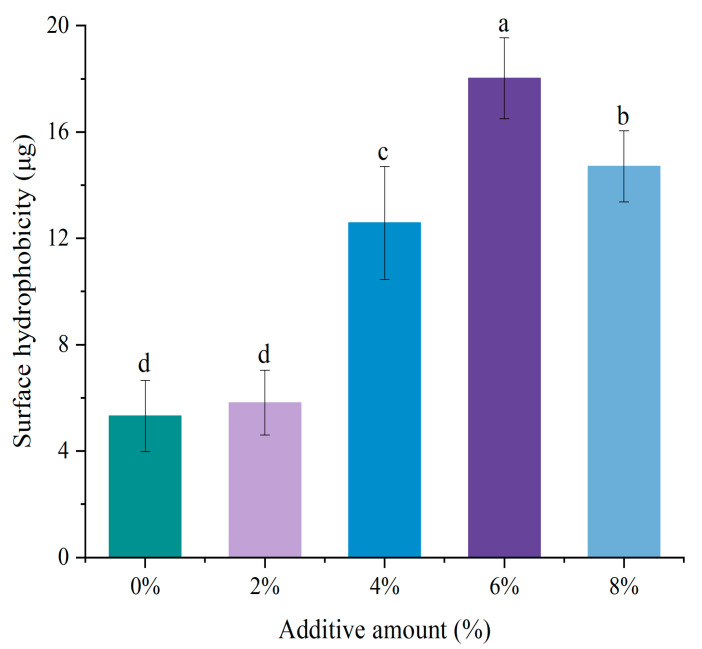
Effect of different DSP additions on the surface hydrophobicity of MP. (a–d) Different letters indicate significant differences within the different addition amounts (*p* < 0.05).

Changes in surface hydrophobicity can reflect the conformation and structure of MP, with higher surface hydrophobicity indicating stronger hydrophobic interactions between protein molecules [27]. The effect of DSP addition on the surface hydrophobicity of MP was shown in Figure 4. As the addition of DSP increased, surface hydrophobicity significantly increased (*p* < 0.05), but the change was not significant (*p* > 0.05) at a 2% addition. Compared to the control group without DSP, surface hydrophobicity increased by 7.26%, 12.70%, and 9.39% at soybean textured protein additions of 4%, 6%, and 8%, respectively. Surface hydrophobicity reached its maximum value when the DSP addition was 6%. The increase in surface hydrophobicity is due to the interaction between soybean textured protein and MP, which may induce partial protein denaturation, thereby improving the functional properties of the protein, such as gelation and WHC [28].

### 3.6. Rheological Properties

**Figure 5:**
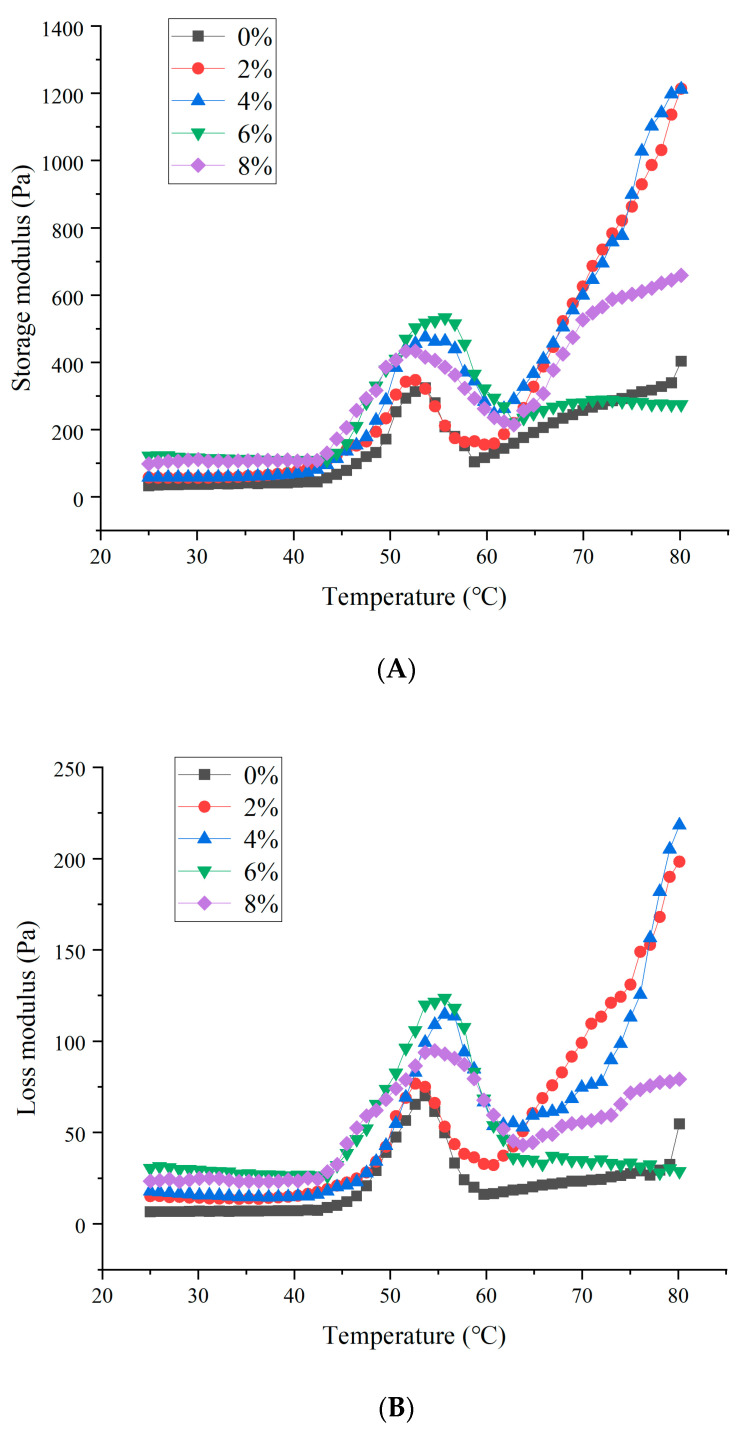
Effect of different DSP addition on the storage modulus (**A**) and loss modulus of MP (**B**).

MP solutions undergo an unstable dynamic rheological process under heating conditions. Studies have shown that the higher the elastic modulus of a substance, the stronger its toughness is, being less flowable and more stable. During frequency changes, the elastic modulus remains essentially constant, indicating a well-formed gel system. Figure 5 illustrated the variation in the storage modulus (G′) of MP with different addition levels of soybean textured protein. As shown, within the temperature range of 20 °C to 80 °C, all treatment groups of MP exhibit a typical G′ curve with three distinct phases. At lower temperature ranges, G′ changes smoothly, indicating that protein interactions are relatively weak at this stage; when the temperature rises to around 43 °C, the storage modulus G′ increases, reaching its peak at around 54 °C. The reason for the difference is the formation of a large number of unfolded MP aggregates in the MP solution after adding varying amounts of soybean textured protein [29], which can enhance the thermal stability of MP and increase the denaturation temperature; further heating leads to a significant decrease in G′, possibly due to protein denaturation resulting in a denser matrix, and when protein interactions occur due to temperature increase, they are compressed and leave some voids, thereby softening; finally, G′ rises rapidly again, possibly due to enhanced protein–protein interactions, which promote the transition from a viscous sol to an elastic gel network [30].

In dynamic rheology, the combination of the elastic modulus and the viscous modulus can determine the transition process from an emulsion to a weak gel and also reflect the degree of molecular cross-linking [31]. The trend of the loss modulus (G″) of MP was similar to that of the storage modulus (G′), but during the heating process, the G′ values are always greater than the G″ values, indicating that MP forms an elastic gel [32]. At lower temperature ranges, G″ changes smoothly. When the temperature rises to around 43 °C, the loss modulus G″ increases rapidly, reaching its peak at around 54 °C; as the temperature further increases, G″ significantly decreases, and finally G″ rises rapidly again. In summary, the sample with 6% soybean textured protein addition has the highest G′ and G″ values, indicating that the viscoelasticity of the MP gel was the best when 6% soybean textured protein was added, a result that was consistent with the results of gel strength and textural properties.

### 3.7. Intermolecular Forces

**Figure 6:**
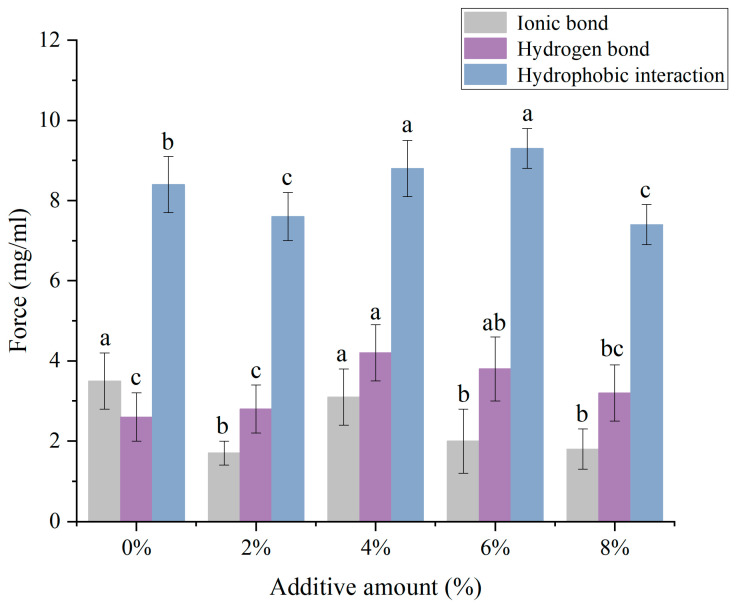
Effect of different DSP additions on the intermolecular forces of MP. (a–c) Different letters indicate significant differences within the different addition amounts (*p* < 0.05).

Figure 6 illustrated the variation in intermolecular forces of protein gels with the addition of soybean textured protein. As could be seen from Figure 6, hydrophobic interactions, which were the main forces maintaining the stability of protein conformation, have the smallest proportion of ionic bonds. With the increase in the addition of soybean textured protein, hydrophobic interactions initially decrease and then increase before decreasing again. The decrease in hydrophobic interactions at 2% and 8% addition levels may be due to the increased viscosity after soybean textured protein absorbs water, restricting the extension of some protein molecules and the exposure of hydrophobic groups within the molecules, as well as the re-burial of exposed hydrophobic groups by an excess of soybean textured protein at 8%. The addition of an appropriate amount of soybean textured protein between 4% and 6% promotes the exposure of hydrophobic groups within MP molecules [33].

Hydrogen bonds, which primarily maintain the secondary structure of proteins, are the main driving force for the transition to β-sheets. The content of hydrogen bonds shows an increasing trend followed by a decrease. The increase in hydrogen bond content may be due to the combination of added soybean textured protein with free water, leading to an enhanced interaction between overall proteins and water molecules. After the addition of 6% soybean textured protein, the decrease in hydrogen bonds may be due to an excess of soybean textured protein competing with MP for water molecules, weakening the interaction between proteins and water molecules, and thus reducing hydrogen bonds [34]. The content of ionic bonds showed a trend of decreasing, increasing, and then decreasing again. The decrease in ionic bond content after adding soybean textured protein may be due to the obstruction of the formation of ionic bonds between proteins and within MP by soybean textured protein, causing protein dispersion. When an excess of soybean textured protein is added, differences in pH values may alter the charge distribution sites of amino acids, leading to a slight increase in ionic bonds [35]. In summary, within the addition range of 0% to 8%, the hydrogen bond values are the highest at 4% addition, and the hydrophobic interaction values are the highest at 6% addition, indicating that the chemical forces of MP gels are most conducive to the stability of protein conformation within the addition range of 4% to 6% soybean textured protein, thereby favoring the improvement of MP gel characteristics.

### 3.8. Secondary Structure

**Figure 7:**
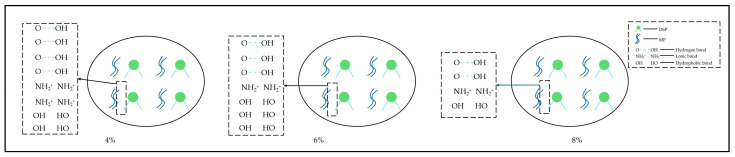
Summary of intermolecular forces.

The relative content of α-helices serves as a benchmark for evaluating the structural stability of proteins. Zhang et al. [36] pointed out that the stability of α-helical structures mainly depends on the hydrogen bonds between the carbonyl oxygen (-CO) and amino hydrogen (-NH) within the polypeptide chain [37]. Figure 7 showed the main secondary structure content of samples with different additions of soybean textured protein. The control group without soybean textured protein had the lowest content of β-turns, at 46.68%, and this content increased with the addition of soybean textured protein. The content of α-helices in all samples was lower than that of the control group, with the highest α-helix content of 27.35% in the control group without soybean textured protein, and this content decreased as the addition of soybean textured protein increased. This reduction in α-helix content indicates that exposed hydrophobic residues cause changes in the structure of MP [3]. Initially, α-helices are first transformed into β-sheet structures, and after a period of oxidation, β-sheet structures are further transformed into β-turn structures [38]. These results suggested that soybean textured protein can prevent structural changes in proteins by enhancing the ordered interactions of MP. Specifically, exposed hydroxyl groups can increase their affinity with amino acid residues, leading to better storage of MP. This observation is consistent with previous research by Gao, Hou, and Zeng [39], which found that soybean polysaccharides and their enzymatic hydrolysis products can reduce freeze-triggered protein denaturation by hindering disulfide bond formation and protein–protein interactions.

### 3.9. SEM

**Figure 8:**
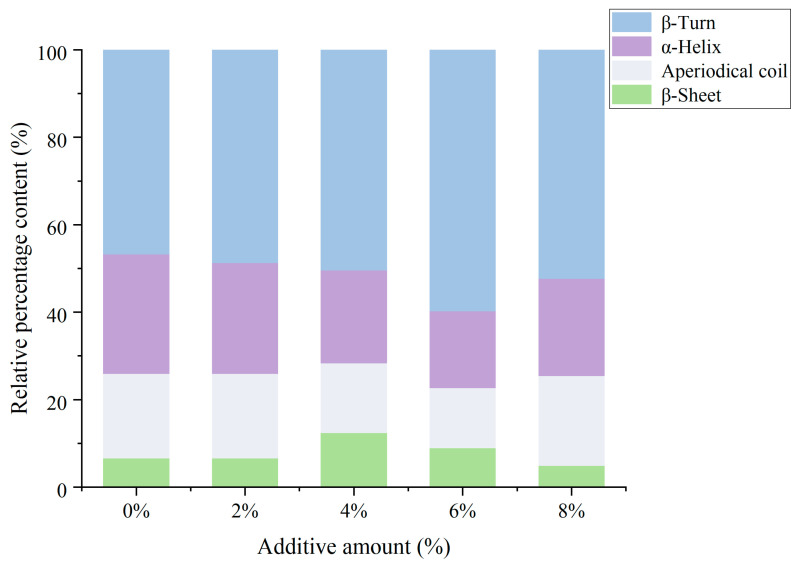
Effect of different DSP additions on the secondary structure of MP.

**Figure 9:**
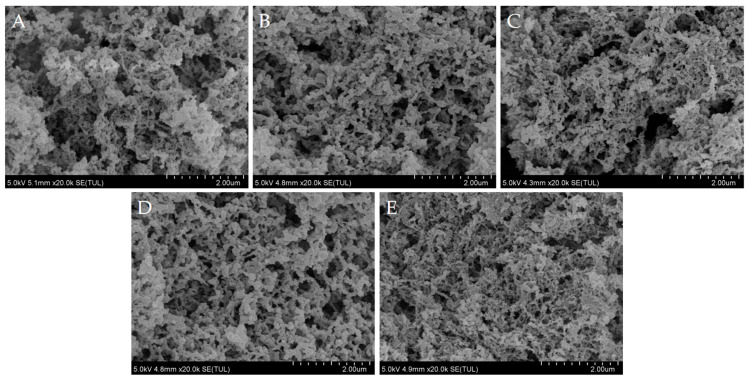
Effect of different DSP additions on the microstructure of myofibrillar protein gel: (**A**), Control group (0% DSP); (**B**), Add 2% DSP; (**C**), Add 4% DSP; (**D**), Add 6% DSP; (**E**), Add 8% DSP.

The visualization of gel structures allows for a more intuitive comparison of interactions, thereby providing new insights into composite gels. Figure 8 and Figure 9 showed the SEM images of MP gels after the addition of soybean textured protein, revealing differences in gel structures formed with varying amounts of soybean textured protein. Among these images, the gel without soy protein shows a loose and disordered state, without a cross-linking effect. The number of pores on its surface is greater, and the pore size is larger. As the addition of soybean textured protein increases, the number of pores on the gel surface decreases, and the structure becomes denser with a more uniform distribution. The further improvement in the microstructure of MP gels may be related to the filling effect of soybean textured protein. Additionally, the incorporation of soybean textured protein promotes hydrophobic interactions between molecules, leading to enhanced gel properties and, consequently, improved gel network structures [40]. Zhao et al. [41] found a correlation between gel strength, water retention, and the spatial network structure of proteins; thus, the microstructure of MP gels is consistent with the results of gel water retention and gel strength.

## 4. Conclusions

This study analyzed the interaction between soybean textured protein (0%, 2%, 4%, 6%, and 8%) and porcine MP and its impact on the gel properties of MP after heating. The results indicated that the addition of soybean textured protein to MP solutions altered non-covalent interactions such as hydrophobic interactions, hydrogen bonds, and disulfide bonds, as well as covalent interactions, affecting protein denaturation and ultimately changing the gel characteristics of porcine MP. When the addition of soybean textured protein was 6%, the interaction between soybean textured protein and MP was the strongest, and the mixed gel formed after heating had the optimal gel properties and a dense and uniform microstructure. Therefore, an appropriate amount of soybean textured protein can effectively induce interactions between MP and soybean textured protein, promote cross-linking and aggregation of protein molecules, form a dense and uniform gel microstructure, and thus improve the gel properties of MP. This study will contribute to enriching the diversity of meat products and providing a theoretical basis for improving the water retention and yield of emulsified meat products in the production process of pork. In the future, it is expected to continue studying the impact of different ratios of soy protein and MP interaction on the gel properties of pork. The research results can further enrich the diversity of meat varieties. In the production process of pork products, they can provide a certain theoretical basis for improving the water retention and output rate of emulsified meat products.

## Figures and Tables

**Table 1:** Effect of different DSP additions on the texture of myofibrillar protein gel.

Index	The Addition Amount of DSP (%)					*p* v.
	0	2	4	6	8	
Hardness (g)	53.62 ± 5.83 ^d^	57.32 ± 4.93 ^c^	65.86 ± 5.19 ^b^	71.48 ± 4.25 ^a^	68.66 ± 6.15 ^ab^	<0.01
Springiness (g.s)	0.51 ± 0.07	0.60 ± 0.13	0.72 ± 0.11	0.74 ± 0.08	0.65 ± 0.09	0.137
Gumminess	5.65 ± 0.79 ^d^	8.09 ± 2.27 ^c^	11.43 ± 2.89 ^ab^	12.3 ± 1.16 ^a^	9.76 ± 1.20 ^bc^	<0.01
Cohesiveness	0.47 ± 0.04	0.53 ± 0.13	0.52 ± 0.06	0.55 ± 0.06	0.45 ± 0.11	0.714
Chewiness	5.32 ± 1.50 ^c^	5.53 ± 1.73 ^c^	8.21 ± 1.08 ^ab^	9.13 ± 2.11 ^a^	6.32 ± 0.89 ^bc^	<0.05
Resilience	0.064 ± 0.013 ^b^	0.079 ± 0.027 ^ab^	0.082 ± 0.021 ^ab^	0.093 ± 0.036 ^a^	0.096 ± 0.028 ^a^	<0.05

Note: (a–d) Indicate that the difference in different addition amounts is significant (*p* < 0.05).

## Data Availability

The original contributions presented in the study are included in the article, and further inquiries can be directed to the corresponding author.
